# Experimental economics for machine learning—a methodological contribution on lie detection

**DOI:** 10.1371/journal.pone.0314806

**Published:** 2024-12-31

**Authors:** Dmitri Bershadskyy, Laslo Dinges, Marc-André Fiedler, Ayoub Al-Hamadi, Nina Ostermaier, Joachim Weimann

**Affiliations:** 1 Faculty of Economics and Management, Otto-von-Guericke University Magdeburg, Magdeburg, Germany; 2 Faculty of Electrical Engineering and Information Technology, Otto-von-Guericke University Magdeburg, Magdeburg, Germany; Uniwersytet Jagiellonski w Krakowie, POLAND

## Abstract

In this paper, we investigate how technology has contributed to experimental economics in the past and illustrate how experimental economics can contribute to technological progress in the future. We argue that with machine learning (ML), a new technology is at hand, where for the first time experimental economics can contribute to enabling substantial improvement of technology. At the same time, ML opens up new questions for experimental research because it can generate previously impossible observations. To demonstrate this, we focus on algorithms trained to detect lies. Such algorithms are of high relevance for research in economics as they deal with the ability to retrieve otherwise private information. We deduce that most of the commonly applied data sets for the training of lie detection algorithms could be improved by applying the toolbox of experimental economics. To illustrate this, we replicate the “lies in disguise-experiment” by Fischbacher and Föllmi-Heusi with a modification regarding monitoring. The modified setup guarantees a certain level of privacy from the experimenter yet allows to record the subjects as they lie to the camera. Despite monitoring, our results indicate the same lying behavior as in the original experiment. Yet, our experiment allows an individual-level analysis of experimental data and the generation of a lie detection algorithm with an accuracy rate of 67%, which we present in this article.

## 1. Introduction

In many experimental disciplines, scientific knowledge, and technological progress are in a reciprocal exchange relationship that significantly accelerates knowledge acquisition and technology development. Findings from basic research lead to the development of new, improved technology, which in turn can be used as an instrument for basic research. This reciprocal acceleration process is well-studied, especially in the natural sciences [1, 2]. In experimental economics research, technology also plays an important role. Note that we focus on experimental economics because of its stricter rules of incentivizing and not deceiving the participants. Yet, most of this argument would be valid for other experimental disciplines like experimental sociology or psychology. From calculators to computer tomographs, technologies of different complexities are used to increase the quality of experiments. However, the relationship between experiments and technology has been a kind of one-way street, because technical innovations are completely exogenous. Experimental economics has made no contribution of its own to the development of better technology. The reciprocal acceleration that we can observe in the natural sciences is therefore not taking place.

The central thesis of this paper is that with machine learning (ML) a new technology is at hand, where for the first time experimental research can contribute to enabling substantial improvement of technology. At the same time, ML opens up new questions for experimental research because it can generate previously impossible observations [3]. Thus, for the first time, there could be a fruitful interaction between technology and experimental economics research.

Despite the high relevance of ML for experimental research [4–6], we do not observe that it is used in experiments to its fullest extent [7]. To understand why this is the case, we first need to clarify which conditions technologies must fulfill to be applied and gain acceptance in experimental research in economics. We argue that a *necessary* condition is that a Pareto improvement is achieved by the use of the technology concerning the three most important quality characteristics of experimental research: internal and external validity and reproducibility. A *sufficient* condition for the use of technology is that it is also available at a low cost, whereby the necessary learning effort for mastering the technology must also be included in the costs. The empirical evidence for these conditions to be decisive is very clear: In the beginning of experimental economics research, it was simple computer networks. Later, mobile devices, smartphones, and the Internet became widely spread. On the other hand, some technological innovations have not managed to get beyond a niche existence. FMRI and eye tracking, for example, have not been able to establish themselves in the same *universal* manner until this day, because they neither could increase the external validity nor were available at low costs. Instead, these are specialized devices applied for specific research questions (e.g., attention, neural activity).

Based on this finding, we argue that the current quality of some ML applications is not high enough to achieve the necessary Pareto improvement. We locate one cause of the insufficient quality in the fact that the datasets needed to train certain ML algorithms are not of the necessary quality. The existence of a ground truth in the data is an important quality criterion in ML. Without knowing the true states it becomes unclear what an algorithm will learn. Yet, for some problems, the knowledge about the real ground truth can only be achieved by conducting controlled experiments. At this point, a door opens for experimental research, allowing it, in turn, to contribute to the advancement of technology. Experimental methodology is extremely well suited for generating data under controlled conditions. It can therefore not only be used to evaluate algorithm-based systems [8, 9] but to generate exactly the data that is best suited to train ML algorithms [10]. In this way, the experimental method can be used to optimize the algorithms, which cannot be achieved based on the so far available data sets. To strengthen the argument, we choose a particularly difficult and relevant case: lie detection. Since the creation of lies is complex [11] and humans cannot detect all the necessary clues of lies [12, 13], lie detection is a hard task and human lie detection accuracies are close to random guesses [14–17]. Therefore, lie detection has become a very promising endeavor in current ML research [18–21] with a potentially large impact on economics and society. Yet, to train such ML algorithms it is necessary to correctly observe and assess lies in the real world in a systematic way. Likewise, from the experimental perspective, lying is a complicated phenomenon to investigate, as anonymity can affect its occurrence [22–24]. Therefore, the experiments used to generate such data sets should be implemented in a trustworthy and transparent way, which is the reason we focus on experimental economics, as this experimental discipline has a long tradition of not deceiving any subjects. In summary, ML algorithms on lie detection benefit from precise experimental data on lying behavior and experimental economics benefits from such ML algorithms by being better able to investigate lying behavior in the experiments.

In this paper, we will use a concrete example (detecting lies using AI) to demonstrate that the availability of datasets for training ML algorithms is indeed the limiting factor for using ML in experimental research (Section 2). We will use the same example to demonstrate how an experiment can be used to generate optimal training datasets, and we will show that doing so can already generate interesting results for experimental research (Section 3).

## 2. Machine learning, lie detection, and the data set problem

### 2.1 A short introduction to machine learning

Machine learning (ML) is a core scientific field of artificial intelligence (AI) that provides machines with the ability to learn without being explicitly programmed [25]. Before ML, AI methods were only strict about solving low-level tasks in business and enterprise settings like automation or rule-based classification. Traditional rule-based methods generate predefined outputs based on particular rules programmed by humans. In contrast, ML models simulate human intelligence by learning the rules from the training data to solve complex problems.

The core concept in ML algorithms is the process of training. The data used for this purpose can be either labeled or unlabeled. Labeled data comes with corresponding ground truths assigning each sample to a certain class or denoting specific information therein. In contrast, unlabeled data comes without any ground truths. Based on the type of data and the way of training, ML distinguishes between supervised and unsupervised learning [26].

Supervised learning using labeled data is the most fundamental type of ML. The ML model tries to find the relationship between input data and ground truths during training to emulate this into a mapping function. Its performance is afterward evaluated on other labeled data, referred to as the test set. Supervised learning is performed when specific goals are identified to be addressed using a certain set of inputs. Thus it represents a task-driven approach [27]. The main drawback of this approach is that labeled data is challenging to obtain.

Unsupervised learning has the advantage of working with unlabeled data to obtain the underlying hidden structure. Hence, it is a data-driven approach [28]. The advantage is that the training data does not need to be extensively prepared by humans. Unsupervised learning models are considered computationally intensive, as they need extensive unlabeled data to output intended results. Their main drawback is that they can provide highly inaccurate results that need to be validated by humans.

For applications that aim to generate outputs for new data solely supervised training approaches can be considered since unsupervised training is only able to determine internal structures and dependencies of an already existing dataset. Due to these constraints concerning possible fields of application, supervised training methods are much more dominant in ML, leading as a consequence to the necessity for annotating the data. During this annotation process, one or more labels are assigned to each image or video sequence, varying in type and quantity depending on the desired task. To accomplish this labeling, one relies on human annotators and experts in the respective field to ensure a high level of data quality.

### 2.2 Lie detection with machine learning

As mentioned earlier, in the following we will use lie detection as an example of an application of ML that can potentially generate an advance in experimental economics research while being improved by experimentally generated datasets. In contrast to other methods to detect lies that require specialized hardware, e.g., eye-trackers [29] or fMRI [30, 31], we focus on a case where no special hardware is needed. ML-based lie detection that works with a normal webcam is more likely to be applied in the real world and thus more externally valid. In principle, ML can detect lies if a lie is expressed in a certain way in the facial expressions of the person who says it. The decisive factor here is that the human face has a great many micro-expressions that cannot normally be consciously controlled. The technology would fail if each person’s facial expressions moved in an individual way during a lie. However, if certain expressions were typical of a lie, these could be detected with a well-trained ML algorithm.

Using a successful lie detection algorithm would mean using a new technology in an economic experiment. But would that represent an advance in experimental research in the sense of the considerations in the introduction? To answer this question, we need to apply the criteria mentioned there.

#### Internal validity

Similar to studies using fMRI technology, ML allows the observation of variables that cannot be observed without this technique. The human eye is unable to detect and correctly interpret microexpressions. Numerous experimental studies on lie detection have shown that humans are very poor at detecting lies [32]. ML can lead to significantly higher probabilities with which lies can be unmasked. Therefore, ML enables experiments that investigate how a technology that can detect lies affects behavior, for example, in negotiations. Will negotiators resort to this technology? How does the ability to detect lies with higher probability change the outcome of negotiations? In summary, it is clear that internal validity increases by such an ML technology for lie detection and that the research horizon is significantly expanded with it.

#### External validity

Concerning external validity, the central question is whether such tools will be applied in the real world. Although forecasts are speculative by nature, there are a few indications that if such technology becomes accessible, it will be applied, as there is already some demand for it in the real world [33]. Assuming the technology will prevail, the application of such a tool in a laboratory experiment increases the external validity.

#### Replicability

Provided that the algorithm for lie detection is available as open source, it should be clear that the replicability of an experiment is given in the same way as it is the case for the replication of standard statistical procedures. Thus, it is clear that ML fulfills the necessary conditions for technical success concerning lie detection because it represents a Pareto improvement concerning the quality criteria considered so far.

#### Costs

Concerning acquisition costs of physical devices, the costs are comparatively low, as normal HD Webcams suffice. Concerning other costs, the ultimate goal of developing such tools in science is to make them publicly available as open source. Yet, by definition, the existence of well-functioning open-source alternatives makes the financial costs for researchers very low. The learning costs are hardly known a priori but the application of the open-source tool is unlikely to be more complicated than currently applied code for statistical analysis in Python, R, or Stata. If commercial software provides an even more intuitive user interface, this will make the application even easier.

The monetary costs and the learning costs are thus so low that they certainly do not stand in the way of widespread use of the new technology. However, one could argue that the use of ML leads to opportunity costs because it is accompanied by a higher control effort and a correspondingly pronounced monitoring of the subjects’ behavior. Behavior is monitored much more intensively than in laboratory experiments without the use of ML, which potentially changes the behavior, e.g., effort or lying behavior [24, 34, 35]. However, at least in the case of lie detection, this corresponds to reality when such technology is used. The awareness of being observed more closely than otherwise is exactly what is needed in terms of higher external validity. In this respect, there are no additional opportunity costs at this point.

Summing up, the application of ML on videos in experimental economics potentially combines some of the benefits of fMRI research (i.e., detecting variables that otherwise remain hidden to humans) with the low costs of common hardware and open-source software. As we discussed, the costs of this technology are moderate. Based on our prior discussion, this means that if the quality of the algorithms is high enough, they are very likely to be applied in experimental economics. This leads to the essential question: how good are these algorithms?

### 2.3 The data set problem

ML has evolved significantly in recent years and in particular, deep learning with its deep neural networks (DNN) has emerged as the fastest-growing field in ML [36]. ML models make decisions based on data fed into them during the training process. From this data, they identify patterns and derive features to perform the subsequent classification. The deeper a network architecture is, the more data is needed for training to adjust the weights according to the given task. The severe drawback of DNNs is that most of them are black boxes [37]. Thus, it is neither transparent for users how and why a decision was made nor for developers what exactly the network has learned. Therefore, the quality of the training data plays a key role, as it is responsible for the later behavior of the system.

In the following, we discuss what is important for high-quality data using the case of video-data-driven lie detection, yet most of the argument is valid for other applications, too. First, we will discuss the data quality parameters of (i) knowing the ground truth and (ii) establishing endogenous lying. Then, we address parameters of (iii) reproducibility of datasets, (iv) deception of subjects, and (v) technological conditions.

First, if it is the central goal to train an algorithm to detect lies, then it is of paramount importance to obtain a data set that contains lies and truths as well as provides the ability for the researchers to distinguish between them with certainty. In the context of this paper, we refer to this as knowing the ground truth. Therefore, when generating data for lie detection algorithms, it is important to obtain full control of the data generation process. This can be done through laboratory experiments. Gathering data in laboratory experiments leads to either fully automatic annotations or annotations that are very easy to conduct by humans. Therefore, this reduces the labor intensity and error-proneness of the data generation and the annotation process.

Second, to establish high external validity of the data set, subjects should lie based on their own decisions in contrast to simply being asked to lie by the experimenter. This resembles much closer to the real world. To make this possible, it is crucial to establish the right financial incentives in the experiment. This can be done by inducing value in the decisions [38, 39]. A carefully controlled laboratory experiment would provide top-quality data, as argued by Marsden and Pingry [10].

The no deception rule applied in experimental economics ensures that subjects trust the experimenter. The importance of the rules is highlighted when considering the opposite. If subjects do not trust the instructions because they are sometimes lied to, their behavior depends on their belief that the instructions are true. This reduces experimental control and therefore, the quality of the data [40].

Finally, when it comes to data quality, it is important to control the technological environment of the experiment. For visual data, in particular, there is a multitude of possible environmental interferences, such as varying illumination and others [41–43]. In addition, camera characteristics can affect the quality [44, 45]. Furthermore, when storing large amounts of data, the type of compression and possible accompanying losses should be taken under advisement [46, 47]. All these aspects are fundamental to the development of sophisticated algorithms capable of dealing with uncontrolled operations [48].

After establishing the quality parameters for high-quality data for lie detection software, we briefly analyze data sets in current use. We identified six major data sets that are used for training lie detection algorithms. Yet, none of these fulfills all the quality parameters we discussed (see Table 1). In some experiments, not even the experimenters know the truth. In others, the subjects are not incentivized to lie but are simply told to do so without any control mechanisms. Further, several experiments employ different types of deception. Thus, while it is possible to train algorithms on these data sets, these algorithms are likely to suffer from the same methodological flaws, inherent to the data generation process. Here, we argue, experimental economics can help. Due to the long history of providing controlled, standardized, well-documented, and incentivized experiments in laboratories with a good no-deception reputation, this methodology can contribute to providing automatically (or easily) annotated data sets for training algorithms. In the following chapter, we provide an example of how a well-studied economic experiment on lying was used to generate such a video data set. Doing so, we show how an experiment from experimental economics can fulfill the above-mentioned quality criteria, i.e., experimenters know the true state of the variables, participants are incentivized (related to the content of the lie as it would be in the real world), and participants are not deceived. However, in the long run, the true potential of using economic experiments would lie in the large number of different methodologically sound experiments that could be used to generate such data sets. Since these experiments aim at investigating isolated elements of lying (e.g. guilt aversion, sizes of stake) they would provide a clearer picture in the data for different types of lying and ML could benefit from knowing these differences for labelling purposes.

**Table 1:** Summary of currently used data sets on lie detection.

Paper	True state objectively known1	Incentivization2	Is incentivization related to the content of the lie	Avoiding subject deception
**DDPM**	No	Yes	No	Yes
**Box of Lies**	Yes	No	No	Yes
**Real-life trial data**	No	Yes	Yes	No subjects
**MU3D**	No	No	No	Yes
**Bag-of-lies**	Yes	No	No	Yes
**Silesian**	Yes	No	No	No

*Note*: the six mentioned data sets come from (in order of appearance): [49–54]

^1^ This refers to some data sets relying on the subjects themselves defining their statements as true or false. Thus, the experimenter does not objectively know the truth.

^2^ Unless clearly stated, we assume no incentivization took place. The real-life trial data constitutes an exemption as people recorded were incentivized through real-life consequences of the judicial system.

## 3. The Experiment

### 3.1 Experimental design

The experiment aimed to observe subjects who, of their own free will, either tell the truth or lie to gain an advantage. To know whether a subject is currently lying or speaking the truth, it is necessary to know the respective "truth." To achieve this goal, we suitably modified the well-known experiment of Fischbacher and Föllmi-Heusi [55] (FFH). We chose this experiment because it is one of the most used designs to investigate lying. Further, it has the advantage of being simple and not involving strategic interactions.

Subjects had to roll a die once. The number of points on the dice is documented on a camera (Camera 1, Logitech HD Pro Webcam C920), which is placed on the left-hand side of their desk. Afterward, they are connected to the experimenter via video chat (using Camera 2, Logitech BRIO 4K UHD) and tell her their respective number of points (Note that the quality of Camera 1 is less important as it only observes the roll of the dice. The quality of Camera 2 is more important. Ideally, it should record at high resolution and high frame rate.). The payoff is based on the number they tell the experimenter into the camera. In line with FFH, for rolling a number between 1 and 5, the subjects receive a payment of 1 to 5€, respectively. If they roll a 6, the payoff is 0€.

**Fig 1:**
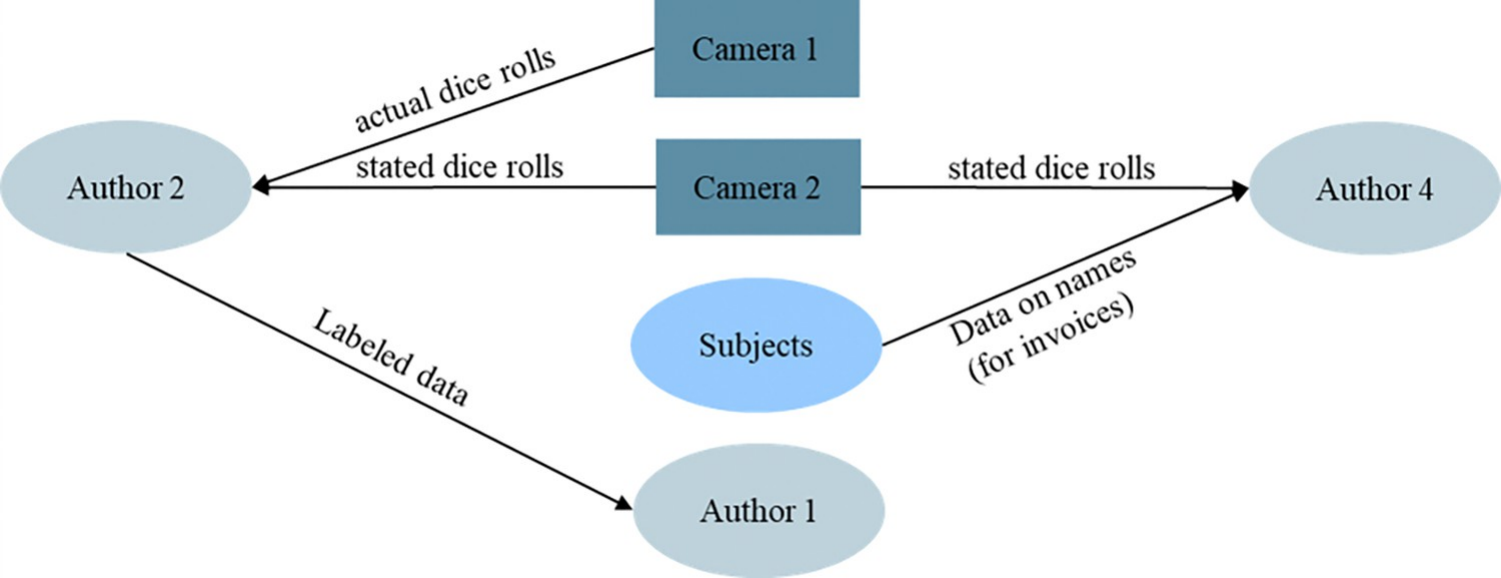
Roles of authors to establish a high level of anonymity for the subjects.

We chose to stick to the original setup and not to use digital dice for two reasons. First, this approach better replicates the original experiment. Second, we aimed to achieve a high level of transparency concerning the monitoring. Concerning the technical setup, Camera 2 is placed on a monitor in front of the subject. It is used for video chat. Camera 1 is downside-faced placed, mounted at a height of 40 cm, and focuses on an area of 20 cm*30 cm where subjects roll a die. Camera 1 is not connected to the experimenter, i.e., she does not check the individual dice rolls and video chat testimonies of experimental subjects. This is important since prior research indicates that the chance of being caught reduces lying behavior [24]. Therefore, it is crucial that the subjects believe this setup. Since Charness et al. [56] observe that a lot of subjects are not aware of the no-deception policy in economics, this rule is explicitly stated and verified by a certificate of the GfeW ethics commission, which is presented to the subjects along with the instructions in the experiment. To further ensure that all our subjects believe this, we excluded all subjects who study psychology from our sample since psychology students conduct a lot of psychology experiments, which can include deception of the subjects followed by a debriefing after the experiment. To ensure the anonymity of data, the research process is split up among the authors, as it is displayed in Fig 1. Author 4 conducted the experiment in the laboratory but was not informed about the true dice rolls. Author 2 received the videos of the dice and annotated them. Using a pseudonymized identifier, Author 2 merged two sets to analyze individual lying behavior, and sent it to Author 1. Author 1 does not have any personal information on the subjects or their videos. This results in a similar situation to the original paper, yet it includes lying information on the individual level.

We used MTVE [57] to conduct and record the video conferences. We recorded the videos at 1280x720 and approximately 60 frames per second (FPS) (Data is stored on the laboratory servers of MaXLab in line with the GDPR (General Data Protection Regulation). In total, we recruited 148 students. We obtained informed written consent from all participants. Out of these, 47 students were in the treatment that directly replicated FFH (without cameras) and 101 students belonged to our variation of FFH (with cameras). The students agreed to be recorded in line with the data regulation of the laboratory. The experiments were conducted between June and October 2022. Each experiment lasted at most 10 minutes. During the sessions, there was no communication between subjects. Because of the COVID-19 pandemic and hygienic regulations, people were told that communication with the experimenter during the session would take place via video chat. The experimenter guided the subjects to their places. Throughout the whole experiment, we used the same two sound-insulated booths with the same LED lighting (Osram LEDinestra 9W). To increase the quality of data, it is useful to keep lighting constant and avoid flickering at the used FPS. At their places, the subjects received instructions (see S1 File) for the experiment. Subjects were fully informed about the experimental setup, there was no deception. We had no issues concerning the compliance of the subjects.

### 3.2 Experimental results

In this section, we will present the results from our experiment, starting with the same type of analysis that was possible in FFH [55]. After that, we will present findings that became possible due to our additional observation.

**Fig 2:**
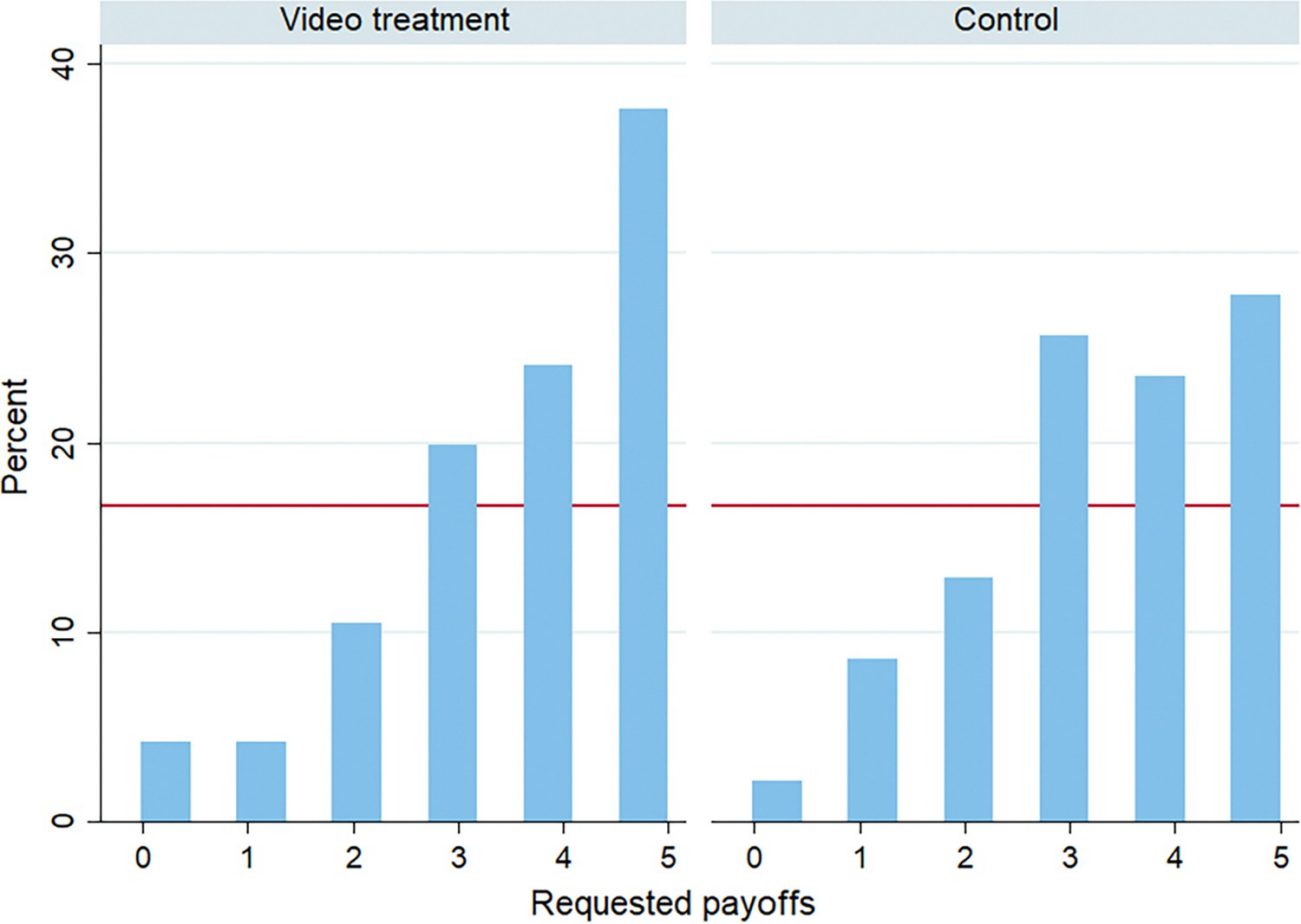
Histogram of requested payoffs in video-treatment and control group. *Note*: The red line represents the 16.67% benchmark which is the probability of rolling one specific number.

After cleaning the data, 96 out of 101 observations can be evaluated. This was necessary because, for some of the videos, it is unclear whether there was a technical issue, the subjects removed the box, or the subjects simply told a number without having rolled the dice at all. Despite this being a very conservative approach, we decided to exclude all of these cases. Looking at the stated dice rolls, we observe the same pattern as in the original paper (see Fig 2). 37.5% of subjects report having rolled a 5 (payoff-maximizing number) and 4.2% report having rolled a six (payoff-minimizing number). To provide additional evidence that this effect is not confounded by a selected sample as compared to the original research, we further conducted a small replication of the original experiment from FFH without monitoring. The results obtained from the control group of 47 students are in line with the original findings and support the argument that the group-level lying behavior did not change due to monitoring. The average requested payoffs were 3.43€ in the control and 3.68€ in the video treatment (MW-Test: p = 0.2224). Likewise, the differences in distribution were also not significant (Kolmogorov-Smirnov: p = 0.987).

Thus, after we indicated that monitoring did not change the results, we focus on the information obtained from the process of labeling the data and investigate individual-level lying behavior. This allows us to distinguish between payoffs that the subjects requested and those they would have deserved due to actual dice rolls. Starting with the information from the labeling, there are two interesting observations. First, two subjects did not roll the dice but visibly put them with their hands such that it showed the number 5. We consider this to qualify as a lie. These subjects were recoded to have deserved a payment of zero euros. Yet, the results do not change if we were to put any other number (e.g., the average payoff of 2.5€) or would have excluded these observations. Second, two subjects rolled more than once to obtain a payoff-increasing number. This is also considered a lie since the subjects were explicitly told to report the number they rolled with their first roll. In these cases, the deserved payment was coded as the payment that would have resulted from the first roll.

On average, subjects requested a payoff of 3.68€, while the actual dice would have yielded a payoff of 2.51€. The difference is highly significant using the Wilcoxon signed-rank test (Bonferroni corrected, p<0.0001). Second, we focus on the question of how many subjects lied. Given our definitions, 37.50% of all subjects lied. Out of 36 individuals who lied to increase their payoff (one individual lied in the other direction), 23 individuals lied to the fullest extent (stating to have rolled a 5). Four subjects lied to state number 3 and nine subjects lied to state number 4. This provides further evidence for partial lying behavior. Further, following the analysis strategy from [57], we can exclude all those subjects who rolled number 5, since they did not have any incentive to lie. This implies that 43.9% of subjects with a financial incentive to lie reported higher numbers than they rolled. All of this information is considered to be unobtainable in the classical setup with anonymity.

As we outlined in this article, in addition to classical experimental results, our experiment led to the generation of a new data set. To the best of our knowledge, we consider this to be the first lie detection video data set that involves incentivized subjects without deceiving them and making labeling the data easy. In short, the data set consists of the videos that remained after removing questionable video files. It provides an opportunity to assess and improve prior prediction models used for lie detection. It is being used in ongoing research, with ML algorithms achieving an accuracy of 67%. In the following chapter, we present some analysis of the dataset. For a more technical description, we refer to [19, 20].

### 3.3 Multimodal approach to lie detection

As we presented above, all available datasets for deception detection are severely limited. Further, signs of deception are often ambiguous, individual, and weakly pronounced, especially in low-stakes contexts. In addition, automatic detection of deception is a complex and highly context-dependent task. For this specific task, techniques such as end-to-end or transfer learning, although effective in other domains, are not optimal. Therefore, we propose a more flexible strategy that is better suited for this case.

**Fig 3:**
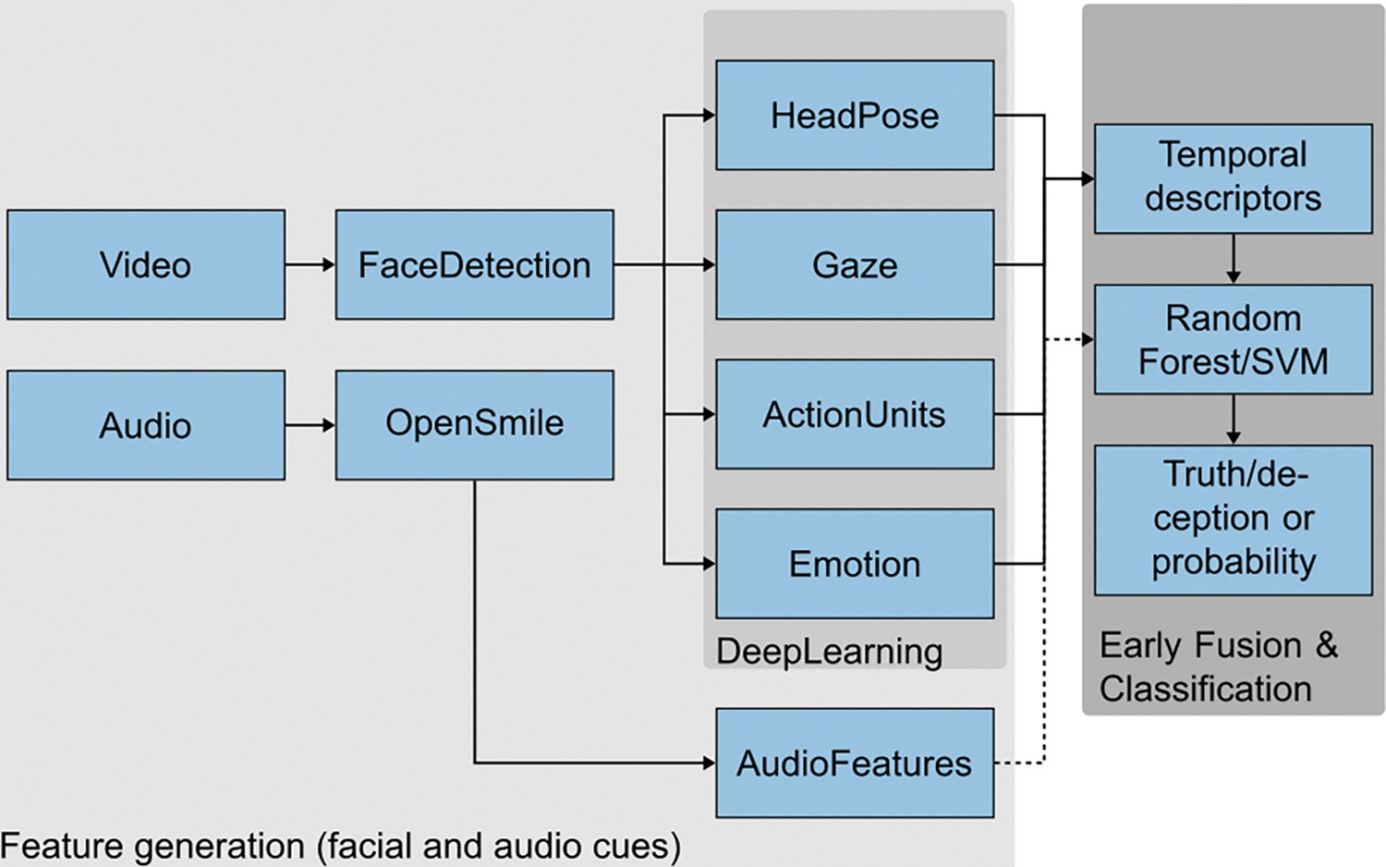
Overview of the proposed approach of automatic detection of lies based on video and audio modalities.

Firstly, various Convolutional Neural Networks (CNNs) are trained to generate different facial cues, such as Action Units (AU) or gaze, as shown in Fig 3. The facial cues are calculated for each frame of a video from which feature vectors for classification are derived through temporal descriptors. The classification can be performed using methods such as Support Vector Machine (SVM) or Random Forest (RF). This constitutes an (early) fusion of individual modalities, which, since the datasets for individual modalities are significantly larger than those for lie detection, works better than a direct deep-learning approach.

**Fig 4:**
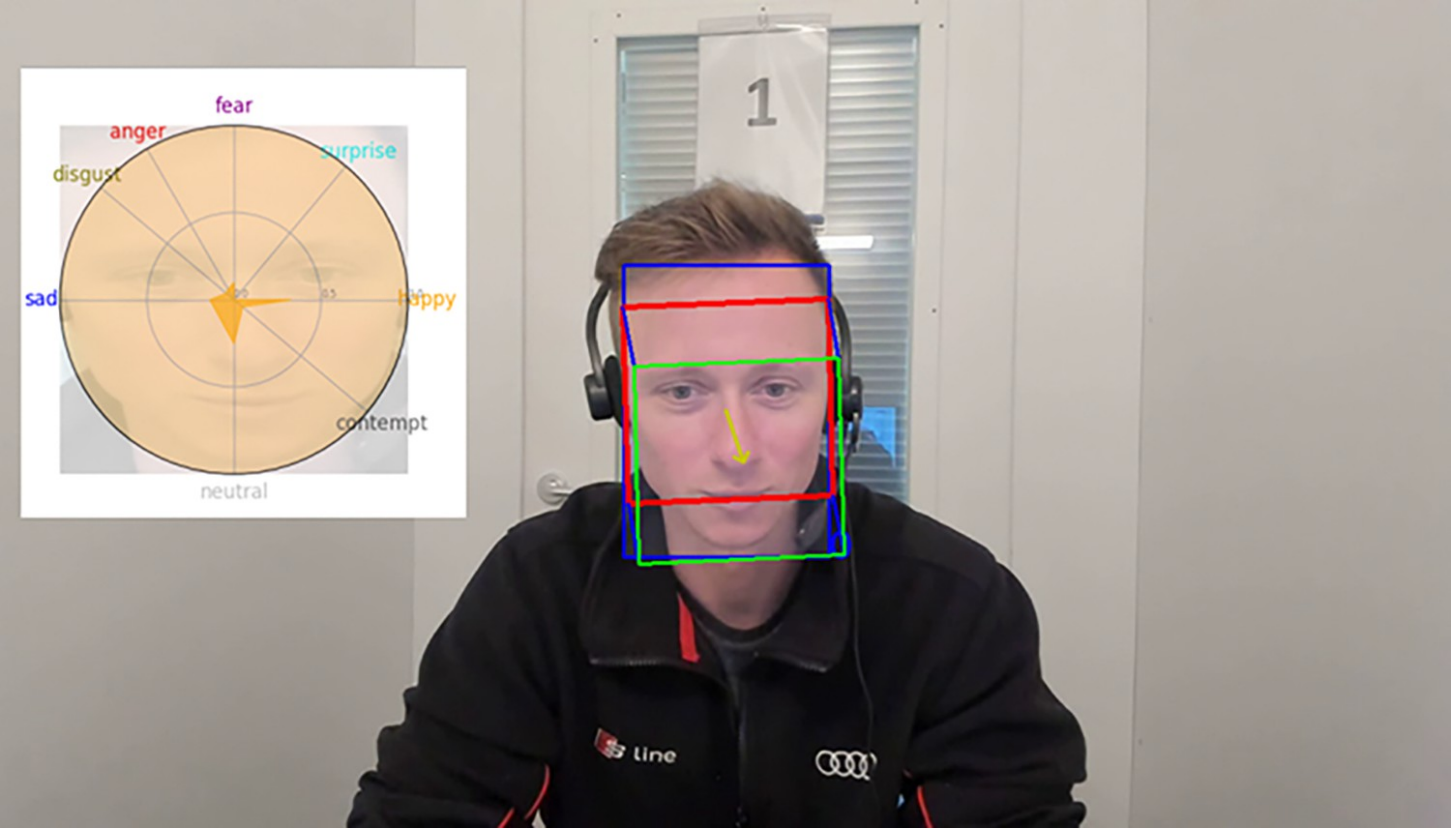
Setup of our Rolling-Dice Experiment and visualization of some of the most relevant facial cues (gaze, head pose, and basic emotions). Note that this scene is not part of the original Experiment due to data protection laws, but the setup is very close.

Fig 4 shows the experimental environment that we have used for the Rolling-Dice Experiment. We visualize some of the facial cues, namely the basic emotions, gaze direction, and head pose. We give further details about the used deep learning approaches in [20].

Our temporal descriptors involve the following: From each time series (where one time series consists of the CNN output for one specific cue for all frames of a sample video), we derive multiple statistics such as minimum, maximum, mean, or skewness. In the future, the existing approach could be expanded by incorporating additional modalities, such as hand gestures or RGB-based vital parameter estimation. However, facial expressions and audio features currently appear to be the most promising for an application that relies on videos from online meetings (which may exhibit a low frequency of hand gestures and suffer from strong compression).

For audio features, we leverage the OpenSmile framework with the extended feature set (eGeMAPSv02). This set derives various features grouped into F1 (openness of the vocal tract), F2 (frontness or backness of the tongue), prosody (rhythm, pitch, and intonation in spoken language), energy, slope, spectral, and Mel Cepstrum coefficients (MfCC).

**Fig 5:**
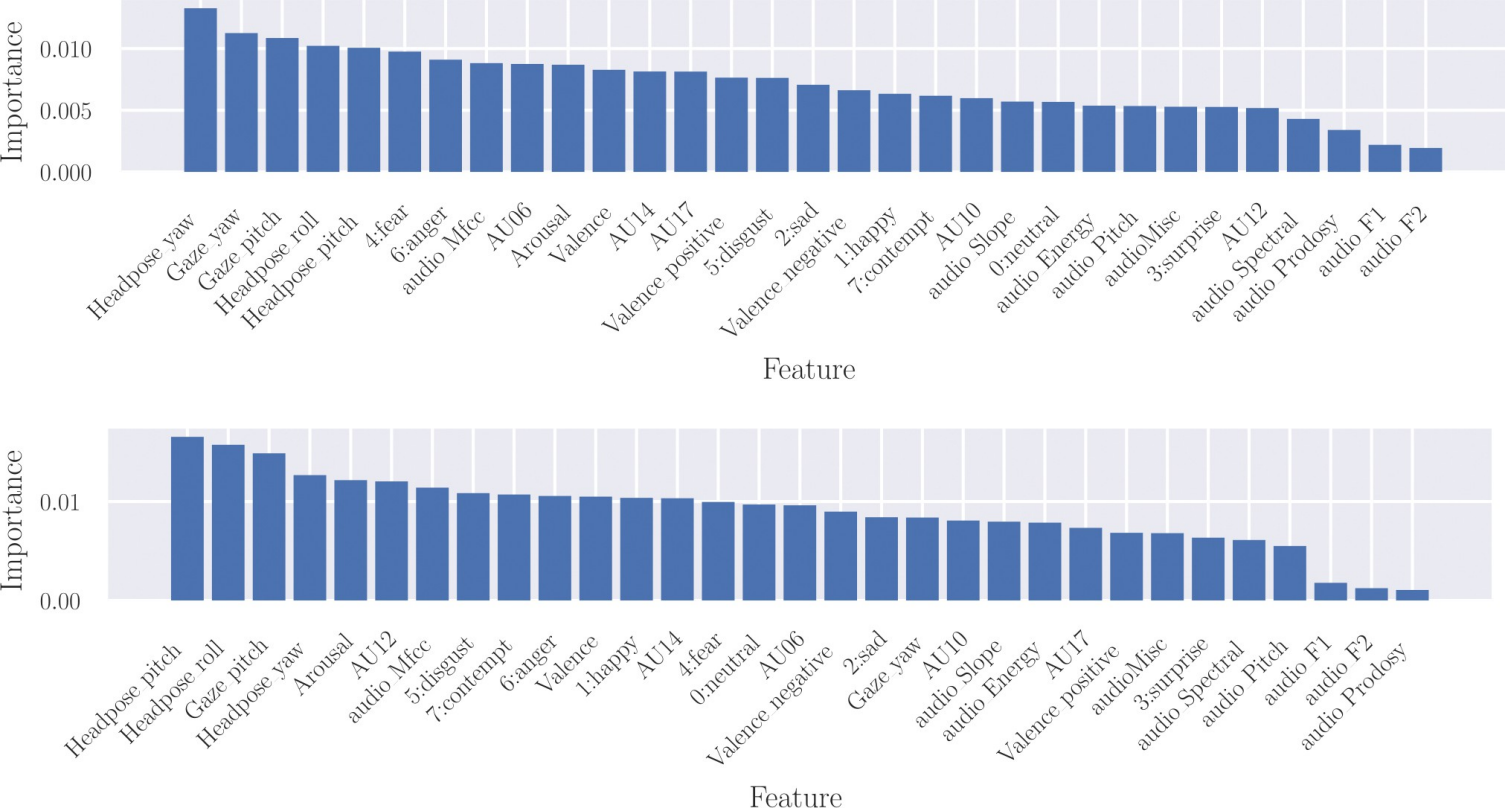
Contribution of all facial cues and groups of audio features to the Real-life trial dataset (top) and our Rolling-Dice Experiment (down).

To assess the contribution of each facial cue and group of audio features to lie detection, we employ the permutation importance technique on both the real-life trial dataset and our Rolling-Dice Experiment (using 70% of the samples for training and the remaining for testing and SVM as a classifier). An interesting finding is that the impact of surprise is weaker compared to that of contempt, as shown in Fig 5. Notably, contempt is not considered a genuine basic emotion, and due to limited training data and facial expressions similar to happiness, it is harder to detect. Since it nevertheless shows a relatively strong contribution, expressions of contempt seem to be a suitable cue of deception that might be missed using only the classical set of the 6 basic emotions. As depicted in Fig 5, certain cues, such as head pose and gaze angles or MfCC features, exhibit a strong contribution in both datasets, whereas the contribution of F1, F2, prosody, or spectral features is notably weak. In general, facial cues seem to be more effective than audio. Nevertheless, some cues, such as AU17 (chin raiser), AU12 (lip corner puller), or gaze_yaw, demonstrate a robust contribution for one dataset but a comparatively weaker influence on the other. Despite results presenting the mean for all subjects in the test set, there exists bias due to the scenario and cultural background. This complicates the training of a system suitable for all scenarios or defining a definitive, universal ranking of cues. This underscores the necessity for a comprehensive dataset tailored specifically to the domain of lie detection in an economic context that can be replicated easily in different countries.

**Fig 6:**
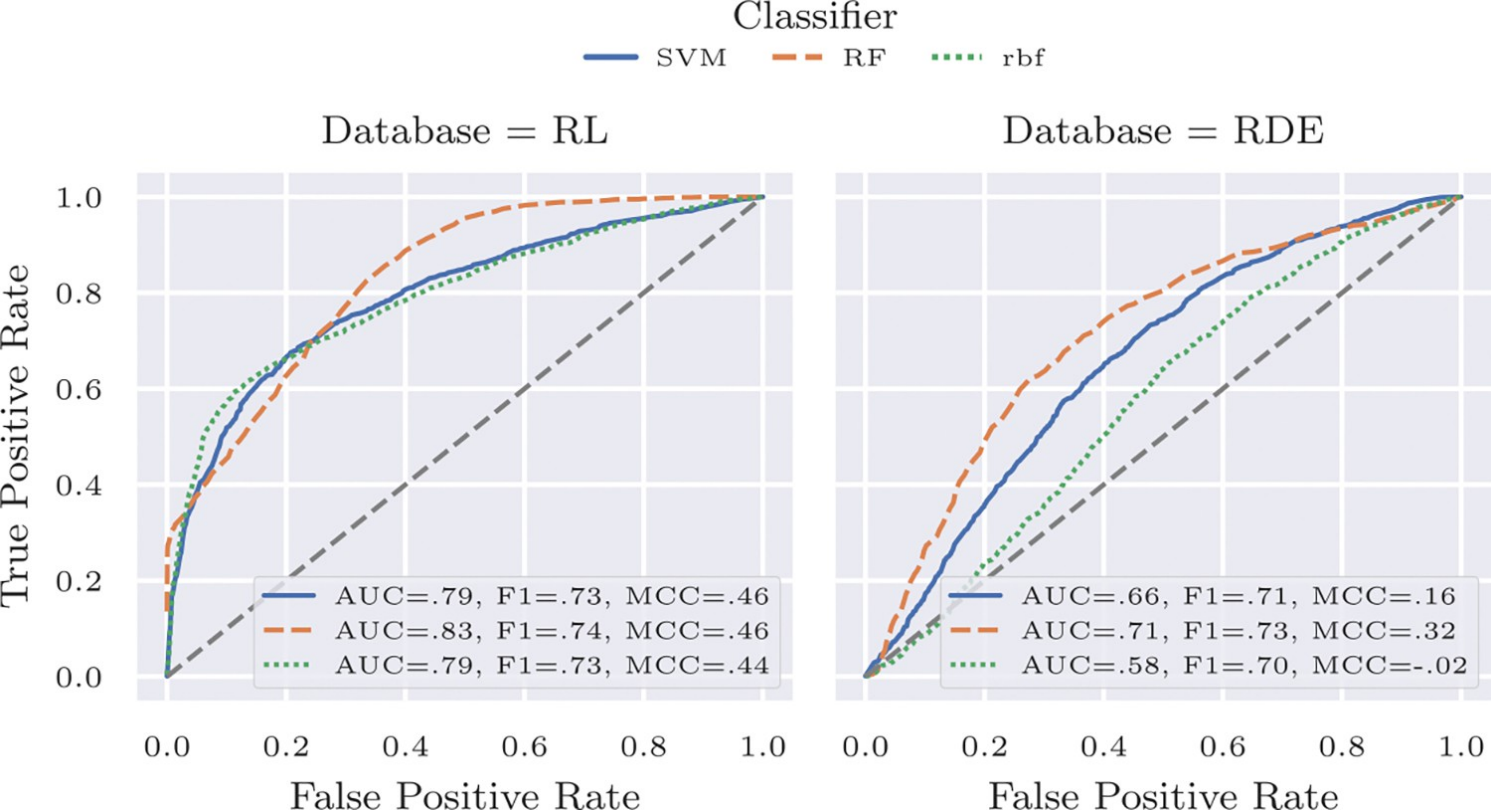
ROC curves for the Real-Life trial dataset (RL) and the Rolling-Dice Experiment (RDE).

Finally, we evaluated our approach on the Real-Life trial datasets (RL) and our Rolling-Dice Experiment (RDE) using stratified k-split (k = 50) and 70% of the samples for training split. Fig 6 shows the ROC curves, which compare three classifiers that are used to fuse the single facial cues and audio features. ROC curves, in simple terms, demonstrate how well a test or model can distinguish between "correct" and "incorrect." The further left the curve is, the less likely there is a false classification of deception (completely excluded at the far left, 0.0). On the Y-axis, you can observe the achievable proportion of detectable correct classifications of deception. This is crucial because, depending on the context, it may be more important to avoid falsely accusing anyone of lying or to overlook as few lies as possible.

The AUC metric summarizes the area under the curve. As depicted in Fig 6, the Random Forest (RF), employing a maximum of 200 trees, slightly outperforms the Support Vector Machine (SVM). It is more evident that lie detection proves significantly more challenging in the RDE compared to the RL dataset. While the number of samples in both datasets is almost the same, the Real-Life Trial dataset involves a high-stakes context and a higher cognitive load, leading to more pronounced reactions and consequently stronger cues for detecting deception. In addition to AUC, we also computed the F1-score as well as the Matthews Correlation Coefficient (MCC) metric (for all metrics, higher values are better). The RDE exhibits a slight class bias, making MCC the preferred measure due to its robustness against such bias. Our results indicate that although it is more challenging to detect lies using only facial cues and audio features in low-stake scenarios (lower MCC values for RDE, especially for SVM), it is possible. Since a lot of human interaction involves low-stakes scenarios, this is an important finding. Still, our analysis indicates that current approaches for automatic lie detection, especially when limited to video and audio data, need improvement for such challenging scenarios. This also involves the necessity of larger datasets to train these approaches. Applying methods from experimental economics will benefit such datasets by enabling replications and (treatment-dependent) variations of the experiment.

**Fig 7:**
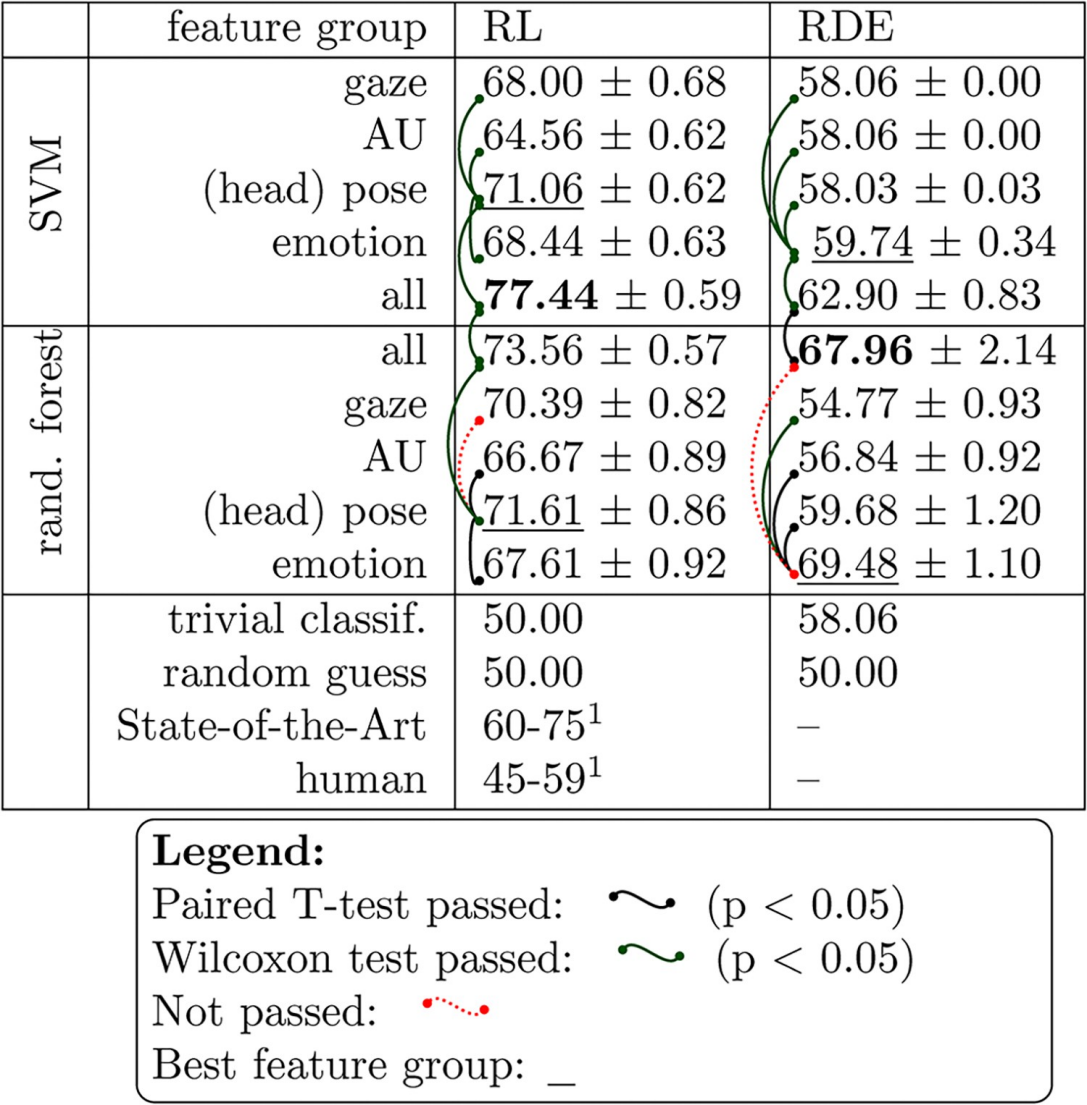
Significance analysis of different feature groups and classifiers on the Real-Life trials dataset (RL) as reported in [51] and the Rolling-Dice Experiment (RDE).

Fig 7 presents the results for single and combined feature sets across the two datasets using SVM and RF. We conducted pairwise t-tests and Wilcoxon tests to evaluate significance, visually represented with connectors. The t-test was applied under the assumption of Gaussian distribution for both variables, with each variable representing the accuracies obtained from the stratified k-split cross-validation for one dataset (including computed mean and standard error). We confirmed the Gaussian characteristic using the Shapiro test and assessed equal variances with the Levene test. This approach enhances the reliability of the t-test, which is optimal for such data. When these assumptions were not met, we employed the Wilcoxon test, which is robust for non-normally distributed data.

We define a group of features from each of the four used CNN models. We conducted pairwise comparisons of the feature group with the highest accuracy against the other feature groups. In the RL dataset, the head pose outperforms the other feature sets. However, in the case of RF, it does not pass the pairwise Wilcoxon test against gaze, so there is no statistically significant difference in accuracy between these two feature groups. For our low-stakes RDE, the emotion feature group is best. This might be due to the setup, as there is no judge whose eye contact a liar might try to excessively avoid (or seek to appear honest). When combining the feature groups into a multimodal classification, accuracy increases. However, for RF classification on the RDE, there is no significant difference in the accuracy of using only emotion or combined feature group. Furthermore, the RF classifier outperforms SVM on the RDE, while the reverse is true for the RL dataset. This discrepancy may be attributed to increased noise resulting from weak or absent reactions in the low-stakes RDE. RF is generally better at handling such noise and extracting complex relationships among features.

We also compare our results for the RL dataset with those of Pérez-Rosas et al. [51]. They employed verbal features alongside nine groups of non-verbal features, such as hand gestures, facial expressions, gaze, and head movements. Using these features, they trained multiple classifiers, achieving a classification rate of up to 75% with all features combined (73.55% for non-verbal features alone). However, these features were carefully handcrafted by experts from the video and audio files of the dataset (not to be confused with manual lie detection). This approach allowed the investigation of various feature groups with reasonable effort, but it does not apply to scenarios requiring real-time or fully automated deception detection. The human accuracy at the bottom of Fig 7 was determined by Pérez-Rosas et al. based on the performance of three annotators, who predicted deception using transcription, audio, video, and combined (video + audio). Their accuracy is notably above the typically expected 52–54% when using video + audio but falls below 50% for video alone. This discrepancy may indicate that subjects reveal more verbal cues in the trial dataset than in other studies that involve human lie detection or that humans are incapable of interpreting facial expressions in the context of deception. However, due to the low number of annotators, no major conclusions can be based on it.

## 4. Ethical considerations

Finally, we want to briefly discuss ethical considerations concerning, first, the data sets used to train ML algorithms on lie detection and second, the application of ML-based lie detection. Starting with the approach to generating datasets, it is important to stress that in generating data containing faces, there are additional problems to those that we have discussed (i.e., knowing the truth, correctly incentivizing lying) in this article. These problems refer to the canonical issue in ML and AI literature–biased data [58, 59] and what can be done to mitigate this problem [60]. Therefore, when generating new datasets, it is important to obtain diverse and representative data sets. Again, experimental economics can help here, as it has rules on publishing the whole experimental procedure such that it can be easily replicated in other countries to create bigger and more diverse data sets.

Second, we briefly discuss what functioning lie detectors would imply for society. Most of the ethical considerations on lie detectors come from the area where it is currently applied the most often–forensics [61]. It is important to stress that such lie detectors usually do not provide evidence beyond a reasonable doubt as would be required in legal terms. Yet, ML-based lie detectors discussed in this article can be applied in all instances when there is a threat of lying, a benefit of catching the lie, and whenever there is a webcam that records the communication process. This broad area implies that many different aspects of our lives (e.g., private communication or job interviews) could be affected by easily applicable lie detectors as these would not require evidence rates beyond a reasonable doubt. The widespread existence of such machines leads to question the importance of (mental) privacy [62] and from an economic perspective the prevalence of false accusations as these could increase due to such algorithms [63, 64]. Both aspects may have substantial effects which could be experimentally analyzed by using prototypes of lie detectors in controlled experiments *before* the widespread application in the real world, thus giving these experiments certain prospective external validity.

## 5. Conclusion

The goal of this article was to investigate whether and how experimental economics can not only benefit from but also simultaneously contribute to the development of certain machine learning algorithms. In so doing, we focused on algorithms designed to detect lies from simple conversational data. Lies are an omnipresent element of our daily lives, daily work, and politics. In bargaining situations, lies can be used to manipulate the other side or to simply keep private information private. A tool that can interfere with these goals in real-time can substantially change the nature of bargaining. Given the amount of literature on the topic of bargaining and private information, these topics are evidently of high relevance. Thus, research on technologies that affect these topics should be of high relevance, too.

Already, these technologies exist. Yet, they rely on disputable data sets. Experimental economics with its rich experimental toolbox can contribute to the development of high-quality data sets that are required to develop good lie-detecting algorithms. Our investigation suggests that, given high-quality algorithms, this technology will fulfill the necessary and sufficient conditions for application in experimental economics. Yet, we conclude that before experimental economics can use such algorithms, it first can support their development. Another beneficial contribution of experimental economics, which was not explicitly discussed in the article, is the analysis of how such ML tools (or their prototypes) will be used by humans. Dealing with technology that has not had a breakthrough in economy and society yet, but is already on the brink, gives experimental research (including experimental economics) certain prospective external validity.

We, further, provided a practical example of how this process can work and can be applied or adapted by other researchers in both fields. We apply a classical experiment on lying from experimental economics (FFH). We modified the design such that it can provide a high-quality video data set on lying. Then, we show that this modification does not affect the experimental results. Instead, it provides a richer statistical analysis on the individual level and contributes to future research ideas (e.g., the role of evasive lies). Finally, we were able to train a lie detection algorithm with an accuracy of 67%. In total, we consider the benefits of such experiments to be substantial. It can deal with classical economic questions on the role of privacy on lying behavior or partial lying and simultaneously contribute to engineering high-quality algorithms by providing the required data sets. Further, following the guidelines of experimental economics, our method enables easy replications, facilitating an expansion of the dataset over different contexts and countries. Our discussion of necessary and sufficient conditions for a technology to be applied in experimental economics indicates that such machine-learning algorithms will be employed in the future. However, to achieve the required quality, there is a need for profound cooperation between experimenters and engineers to provide the high-quality data necessary to improve the algorithms.

## Supporting information

S1 File Translated instructions. (DOCX)
