# Supplementary material for: Experimental economics for machine learning—a methodological contribution on lie detection
Source: PLoS One. 2024 Dec 31;19(12):e0314806. doi: 10.1371/journal.pone.0314806 (PMC11687750; doi:10.1371/journal.pone.0314806)
Supplement: S1 File — (DOCX) [file pone.0314806.s001.docx]

# Supporting Information

# S1. Instructions

## Translated Instructions: Camera-treatment

Ethical review by the Society for Experimental Economic Research (GfeW)

The GfeW with identification code (fnax4PA2) certified this experiment. This is a confirmation that all the information in the instructions is true. A copy of the audit is enclosed.

Instructions

For the following experiment, you will receive a payout that depends on the number of points you score with a single dice roll.

To comply with hygiene measures, communication in this experiment will be via the screen. You can now take off the mask for this. For communication, please put on the headset in front of you.

Your task is to throw a fair die **once** into the marked area to your left. The die may only be rolled once and must be within the marked area.

After you have rolled the dice, you can click on the "Next" button and you will be connected to the experimenter via video chat. She will ask you what number you rolled. Please answer in a complete sentence:

I rolled the number X.

Your payoff depends only on the number of dots you speak into the camera. The experimenter does **not** know what number you actually rolled. Even after the experiment, she will not know.

The following table shows you the payout for each number rolled:

| Number of eyes on dice | 1 | 2 | 3 | 4 | 5 | 6 |
| --- | --- | --- | --- | --- | --- | --- |
| Payoff | 1€ | 2€ | 3€ | 4€ | 5€ | 0€ |

Say you rolled a number between 1 and 5, your payout is €1 to €5 accordingly. If you roll a 6, your payout is 0€.

Then, when prompted, you can get up and collect the corresponding money from the experimenter and leave the lab. To do this, put the mask back on.

From now on, please follow the instructions on the screen.

## Translated Instructions: Control group

For the following experiment you will receive a payoff that depends on the number of points you score with a single roll of the dice.

Your task is to roll a fair die **once** in the marked area to your left. The die may only be rolled once and must be within the marked area. You have 30 seconds to roll the dice.

Your payoff depends only on the number of points you enter in the input field. The experimenter does **not** know what number you actually rolled. Even after the experiment, she will not know.

The following table shows you the payoff for each number rolled:

| Number of eyes on dice | 1 | 2 | 3 | 4 | 5 | 6 |
| --- | --- | --- | --- | --- | --- | --- |
| Payoff | 1€ | 2€ | 3€ | 4€ | 5€ | 0€ |

Say you rolled a number between 1 and 5, your payoff will be €1 to €5 accordingly. If you roll a 6, your payoff is €0.

After you have rolled the dice, please click on "Next" and enter your rolled number. Once the experimenter has called your booth number, please step out of the booth. You can then collect your payoff and the experiment is finished after that.
